# Few Ant Species Play a Central Role Linking Different Plant Resources in a Network in Rupestrian Grasslands

**DOI:** 10.1371/journal.pone.0167161

**Published:** 2016-12-02

**Authors:** Fernanda V. Costa, Marco A. R. Mello, Judith L. Bronstein, Tadeu J. Guerra, Renata L. Muylaert, Alice C. Leite, Frederico S. Neves

**Affiliations:** 1 Graduate School in Ecology, Conservation, and Wildlife Management, Federal University of Minas Gerais, Minas Gerais, Brazil; 2 Department of Ecology and Evolutionary Biology, University of Arizona, Tucson, Arizona, United States of America; 3 Department of Botany, Federal University of Minas Gerais, Minas Gerais, Brazil; 4 Department of Ecology, São Paulo State University (UNESP), São Paulo, Brazil; Universidade de Sao Paulo Faculdade de Filosofia Ciencias e Letras de Ribeirao Preto, BRAZIL

## Abstract

Ant-plant associations are an outstanding model to study the entangled ecological interactions that structure communities. However, most studies of plant-animal networks focus on only one type of resource that mediates these interactions (e.g, nectar or fruits), leading to a biased understanding of community structure. New approaches, however, have made possible to study several interaction types simultaneously through multilayer networks models. Here, we use this approach to ask whether the structural patterns described to date for ant-plant networks hold when multiple interactions with plant-derived food rewards are considered. We tested whether networks characterized by different resource types differ in specialization and resource partitioning among ants, and whether the identity of the core ant species is similar among resource types. We monitored ant interactions with extrafloral nectaries, flowers, and fruits, as well as trophobiont hemipterans feeding on plants, for one year, in seven rupestrian grassland (*campo rupestre*) sites in southeastern Brazil. We found a highly tangled ant-plant network in which plants offering different resource types are connected by a few central ant species. The multilayer network had low modularity and specialization, but ant specialization and niche overlap differed according to the type of resource used. Beyond detecting structural differences across networks, our study demonstrates empirically that the core of most central ant species is similar across them. We suggest that foraging strategies of ant species, such as massive recruitment, may determine specialization and resource partitioning in ant-plant interactions. As this core of ant species is involved in multiple ecosystem functions, it may drive the diversity and evolution of the entire *campo rupestre* community.

## Introduction

Animals and plants live in a “tangled bank” of interactions [1], a network formed by different types of positive, negative, and neutral associations [2]. The nature of these multispecies systems has been illuminated by theoretical advances in community ecology [3]. Network analytical tools have allowed the operationalization of ecological concepts such as specialization [4], functional groups [5], and keystone species [6]. However, despite this boom in the field, studies of most plant-animal networks focus on a single interaction type at a time, or interactions mediated only by a single type of resource. A very few studies have attempted to model antagonistic and mutualistic interactions in the same network, but most of them without empirical data [7], and using simulation models to understand interactions structure [8,9]. To our knowledge, only one empirical study has explored how certain species have dual roles, acting both as seed dispersers, as seed predators in a network [10]. This dominant approach hinders further developments [11], especially considering that individual species are involved in many kinds of interactions at the same time [12].

Ant-plant associations are an outstanding model to study complex ecological interactions, as ants can play distinct functional roles simultaneously [13]. Many ants are considered herbivorous, with most of their food coming directly or indirectly from plants [14]. Most well-studied interactions between ants and plants are putatively mutualistic, with plants providing shelter (e.g., nesting cavities) and food (e.g., extrafloral and floral nectar, pollen, food bodies, and fruit pulp and exudates), and ants providing diverse benefits in return, including protection against natural enemies [15], seed dispersal [16], and even pollination [17]. Another widespread resource indirectly provided by plants is honeydew, an exudate rich in carbohydrates and amino acids, which is excreted by certain hemipteran insects that feed upon the phloem of several plant families [18]. In this association, the ants feed on honeydew, while the hemipterans, termed trophobionts, gain protection from their natural enemies [18]. Although ant-trophobiont interactions are antagonistic to the plants on which they occur, when ants tend trophobionts, they might have ecological and evolutionary impacts on plant fitness [19]. Ants in turn might gain some benefits from these interactions, as has been demonstrated by higher abundance of pupae in ant colonies supplemented with elaiosomes [20] and higher growth and survivorship when workers fed upon EFNs [21] or trophobiont secretions [22].

Ant-plant interactions are mediated by plant-derived food rewards that vary in quality [23], predictability, and availability in the environment [24]. These factors may influence ant behavior and foraging strategies, leading to differences in the structure of interaction networks according to resource type. In fact, recent findings have shown that ant dominance over resource usage is the main mechanism responsible for differences in specialization of networks formed by interactions between ants and extrafloral nectary (EFN)-bearing plants, and between ants and honeydew-producing hemipterans [25]. Likewise, results from compiled datasets suggest that ant-flower networks are more specialized (i.e., more modular) than ant–Hemiptera and ant-EFN networks [26].

More broadly, evidence indicates that mutualistic networks have in common a fundamental property: the presence of a core formed by the most influential species, which reach high scores of centrality [27]. In ant-plant networks specifically, is known that a few central ant species form a core that strongly influences the structure of the entire community [28]; this is especially true in generalized ant-EFN networks compared to those involving specialized myrmecophytic plants [29]. This core of central ant species is consistent in space [28] and time [30], and consists mostly of dominant species displaying high recruitment rates and strong territoriality [31].

These findings have brought important insights to the understanding of ant-plant networks. However, any one ant species uses multiple kinds of plant-derived resources at a single time [14]. It is not known whether a single ant species plays a different role in the community according to the type of resource it collects. Thus, we need empirical studies that integrate different interactions into a complete ant-plant network, in order to understand plant resouce use by foliage-dwelling ants. New models of multilayer networks have recently opened the possibility of studying several interaction types simultaneously. In multilayer networks, interactions between species may be of two or more types, creating interconnected layers [32]. This breakthrough allows us to address a new question: do the structural patterns described to date for ant-plant networks hold when interactions with different resource types are considered? To investigate this issue, we studied one multilayer network formed by interactions between ants and a set of plants that provide different food rewards (EFNs, flowers, fruits), and that also host trophobionts, another food source. We tested whether networks formed by interactions between ants and different food types differ from one another in specialization and resource partitioning among ants. In addition, we tested whether the core of central ant species is similar among resource types.

## Materials and Methods

### Study area

The study was carried out in Morro da Pedreira Environmental Protection Area, the buffer zone of Serra do Cipó National Park, in the southern region of the Espinhaço Mountain Range, southeastern Brazil (19°17'27.3" S, 43°35'40.8" W). We studied ant-plant interactions in rupestrian grasslands, or *campo rupestre*, a megadiverse mountainous ecosystem composed of grasslands and rocky outcrops occurring mainly from 900 to over 2000 m asl. in Brazil [33,34]. It is characterized by a species-rich vegetation, high levels of plant endemism, and a large number of threatened plant species [35]. *Campo rupestre* are also characterized by high ant species richness (288 species), with the highest diversity found in the Cipó Mountains [36]. The vegetation is comprised mostly of small sclerophyllous evergreen shrubs and herbs associated with rock outcrops within quartzitic and sandstone soils with high levels of aluminum and low concentration of nutrients [35]. The climatic regime of this region is characterized as tropical altitudinal (Cwb) according to Köppen’s classification [37], comprising markedly dry and cold winters and hot and wet summers, with mean temperature around 22°C and mean annual rainfall of 1,500 mm [33]. All permissions to visit and collect biological data were authorized by ICMBio of the Brazilian Ministry of Environment (SISBIO authorization number 38952–6). Data collection in sites located at private lands was authorized by the owners and ICMBio.

### Sampling design

We selected seven sites similar to one another in altitudinal range (from 1100 to 1200 m asl.), climate regime, and plant species richness, but distant by at least 1.44 km from one another. We chose these sites not for comparative purposes but in order to capture a representative sample of the area. At each site, we delimited one transect 200 m in length and 1 m in width, which was divided into 20 plots (10 x 1 m). We randomly sampled five plots at least 30 m away from one another. In each plot, we marked all trees, shrubs, subshrubs, rosettes and herbs that were fully accessible to us, those 50–200 cm in height.

### Assessment of ant-plant interactions

We monitored the marked plants quarterly in 2014, at the peak and at the end of the rainy and dry seasons (respectively, January, April, July, and October). Between 0800–1200 and 1400–1700, each plant was observed for approximately 3 min. The interaction event was recorded only when the ant was observed feeding upon the food source [38,39]. We computed interaction frequency when we observed the same pair of species interacting in a different event. We also recorded the number of worker ants using the resource at the time of monitoring to estimate the recruitment rate of each ant species.

We classified interaction events according to the type of resource used by ants: extrafloral nectar and similar secretions (EFNs), floral nectar or pollen (flowers), glands and fleshy pulp of fruits (fruits), and honeydew droplets from trophobiont hemipterans (trophobionts). When we observed an ant on an individual plant that did not provide any resource, or an ant that left a plant without making contact with resources of any type, we defined the interaction as a “visit”.

We collected vouchers of plants and insects for taxonomic identification. To identify ants we used the key by Baccaro et al. [40] and also consulted a specialist. We deposited ant vouchers in the entomological collection Padre Jesus Santiago Moure at the Federal University of Paraná (UFPR). Trophobiont insects were identified using the key by Rafael et al. [41], and also by consulting experts. Vouchers are deposited in the collection of the Insect Ecology Lab at the Federal University of Minas Gerais (UFMG). We identified plants with the support of botanists from UFMG, and deposited vouchers in the herbarium of the Botanical Department (UFMG).

### Network structure

We built a multilayer network formed by ants and their interactions with EFNs, flowers, fruits, trophobionts, as well as visit events, from five weighted matrices, with plant species as rows and ant species as columns, and cells filled with the number of interaction events of that type observed between a *i* plant species and a *j* ant species. We built one matrix for each food type incorporating interactions recorded over the entire year across the seven sites, each representing one layer of the network. Trophobiont associations with ants were analyzed from an ant-plant perspective, so the respective matrix was built only with ant and plant species connected to one another through trophobionts. Since most studies have explored trophobiosis from the trophobiont-ant viewpoint, we choose this approach in order to bring insights for indirect effects of ants on plants (see [42] for a similar approach). Moreover, in the focal habitat, plant-trophobiont interactions are very specialized and modular (S3 Table), making this approach useful to compare ways in which ants are associated with plants. Thus, the multilayer network comprised all types of events recorded in the seven sites during one year of sampling. For some analyses, we assessed the interactions as a multilayer network, while for other analyses, the data were divided by food type into five layers.

To test whether different resource types are associated with different patterns of specialization and resource partitioning by ants, we chose four network metrics frequently used for this purpose: nestedness, modularity, complementary specialization, and niche overlap. Those metrics have the additional advantage of being insensitive or only moderately sensitive to sampling completeness and network size [43]. We computed those metrics for the multilayer network and for each layer separately. Nestedness may provide additional insights into feeding preferences, as in a nested network, interactions involving the least-connected species are a subset of the interactions made by species in the core [44]. We evaluate nestedness using the WNODF metric, which is based on overlap and decreasing fill in the weighted matrix [45].

Network modularity is used to assess whether some groups of species are more densely connected to one another than to other species within the same network [46]. Modularity is positively correlated to network specificity, because distinct modules require a certain degree of specificity in the community, and thus can be used as a proxy of specialization [47,48]. We calculated modularity using the QuanBiMo algorithm, which was developed specifically for weighted bipartite networks [48] and is based on a simulated annealing approach. The level of modularity (Q) measures the extent to which species interact mainly with other species of its own or other modules, and ranges from 0 to 1. Since the algorithm is stochastic, module arrangement can vary between iterations. For this reason, we retained the optimum Q value as being the highest value after 1,000 iterations. Values of Q were standardized (standardized Q), considering the number of standard deviations above the average value recorded in 1,000 iterations. Thus, values of standardized Q indicate significant values of modularity, since they represent how many standard deviations the real Q-value is far from the mean of 1,000 Q-values generated from randomized networks using the QuanBiMo algorithm [48]. Therefore, instead of P-values, we used standardized Q-values to estimate the significance of modularity.

Complementary specialization (H_2_’) was derived from Shannon entropy and describes interaction diversity, i.e., how evenly distributed the weighted interactions are in a network. This index is very robust to differences in sampling effort and network size [43]. Values closer to 0 indicate high generalization or redundancy of interactions, and values closer to 1 indicate high specialization [29,49].

Niche overlap among ant species was also calculated using the Morisita-Horn index, which varies from 0 to 1 [50]. We used the Patefield null model to estimate the significance of the observed network metrics and expectations from 999 randomized networks [51]. All network metrics and their significances were calculated in the bipartite (Dormann et al. 2008) and vegan [52] packages for R [53].

### Differences among resource types

To test whether network metrics vary with food type, we compared network metrics between pairs of resource layers: ant-EFN *vs*. ant-trophobiont, ant-EFN *vs*. ant-flower, and ant-trophobiont *vs*. ant-flower. Ant-fruit interactions were removed from the statistical analysis because the networks formed by them were too small (S1 Table). For this reason, several metrics could not be reliably compared, as they are strongly biased by network size [43]. In this analysis, we pooled the seven sites in order to increase the robustness of the network analysis. We calculated the pairwise differences between layers, then tested whether the observed differences were lower or higher than expected by chance using a Monte Carlo procedure with 999 randomizations of pairwise differences (α = 5%).

To explore differences in general descriptors of network structure among resource types, we computed network size (i.e., number of interacting species), frequency of interactions, and richness of interactions for each layer considering the site as a sampling unit (n = 7 sites). We calculated generalized linear models (GLMs) in which network descriptors (size, richness and frequency of interactions) were the dependent variables and resource type (EFNs, trophobionts and flowers) was the predictor variable. GLMs were compared with null models, and the residuals were analyzed to verify the suitability of the models based on the Poisson distribution of errors.

### The core formed by central species

The concept of centrality is useful to assess the relative importance of a species to the structure of the whole network [54]. There are several centrality indices proposed in the literature [28,55], most of which can be used to determine a core/periphery structure in a network. Degree centrality is the simplest, as it is measured as the number of connections (links) made by each species (nodes) [6,56]. We chose degree centrality to identify the core of central species in each resource layer so that we could test whether this core is similar across them. For each resource type, within each sampling site (n = 7) we selected the ant and plant species with degree centrality above the network average. We then calculated a permutational multivariate analysis of variance (PERMANOVA, [57]) to test whether this core of central ant and plant species is similar across resource types, and ordered the layers with nonmetric multidimensional scaling (NMDS). These analyses were made in the package vegan for R [53].

All statistical analyses were performed in R 3.2.3 [53], and network drawings were prepared in Pajek 4.09 [58].

## Results

### Species and their interactions

We monitored a total of 1,114 individual plants from 108 species and 32 families. The most represented families were Asteraceae (28% of sampled plants), Velloziaceae (12%), Malpighiaceae and Melastomataceae (8% each), Lythraceae (6%) and Fabaceae (5%). In general, the sampled vegetation was 80 ± 29 cm in height (mean ± SD) and was composed mainly of subshrubs (46% of plants), shrubs (36%), rosettes (12%), trees (5%), and herbs (1%).

**Fig 1:**
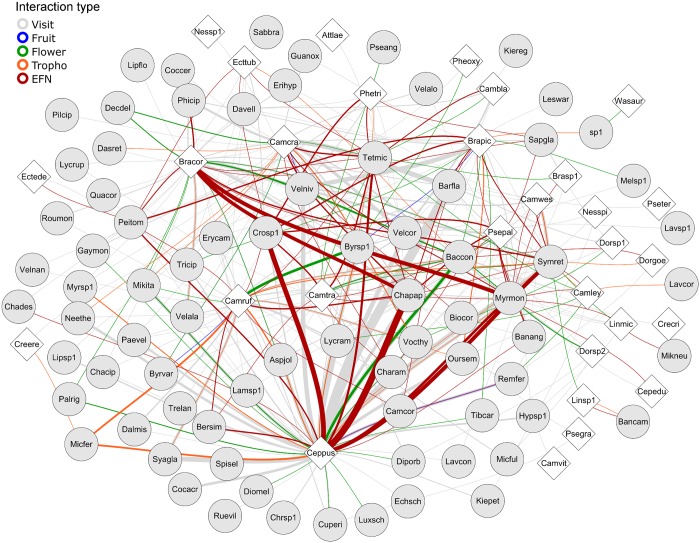
Multilayer network formed by interactions between ants and plants that provide different food types. Circles represent plant species and diamonds represent ant species. Lines represent interactions and line thickness is proportional to interaction frequency. Line color represents the type of resource used. See ant and plant species names in S1 and S2 Tables, respectively.

The multilayer network comprised 795 interaction events between 78 plant species and 30 ant species (Fig 1). Hence, 30 plant species were either not visited by ants or else lacked EFNs and other food sources used by ants (S1 Table). Sixty-six percent of all events were considered visits (i.e., ants were observed on a plant but were not seen feeding on it), 20% involved ants feeding on EFNs and similar secretory structures, 8% involved ants feeding on flowers (nectar or pollen), 5% involved ants tending trophobionts, and 1% involved ants feeding on fruits (eating the pulp or feeding on fruit secretory structures) (Fig 1).

We observed 1,770 ant workers of five subfamilies and 30 species. Of these, 18 species fed on EFNs, 17 species fed on flowers, 12 species fed on trophobionts, and four species fed on fruits. *Cephalotes pusillus* (37% of all records), *Brachymyrmex cordemoyi* (12%) *Camponotus rufipes* (10%), *Camponotus crassus* (10%), and *Brachymyrmex pictus* (6%) together made up 75% of all records and were observed interacting with the most types of food (except fruits). The ant-EFN and ant-flower layers shared 12 ant species, the ant-EFN and ant-trophobiont layers shared 11, and the ant-flower and ant-trophobiont layers shared eight ant species. All ant species in the ant-fruit layer were found on additional resource layers as well.

Visits were made to 71 plant species, among which the most important species were those that provided nesting sites (e.g., *Vellozia* spp.) and EFNs (when this structure was not active). Plant species with EFNs were the most represented in the multilayer network (18%, n = 14, S1 Table). The families with EFN-bearing species were Fabaceae (5 spp.), Malpighiaceae (3 spp.), Euphorbiaceae (3 spp.), Myrsinaceae (1 spp.), Araceae (1 sp.), and Polygonaceae (1 sp.). Three Asteraceae species (*Baccharis concinna*, *Symphyopappus reticulatus*, and *Mikania neurocaula*) secreted other substances within their leaf blades, probably resins, which the ants collected. Since ant behavior when attending these structures was similar as in the case of EFNs, we pooled these interaction types for a total of 17 species with secretory structures in the multilayer network (22%, 11 exclusive species, S1 Table). Ants used flower resources on 23% of plant species (23 spp., S1 Table). Of those species, 14 were exclusive to the flower layer. Interactions with fruits were observed only between four plant species (5% of all species) and four ant species (S1 and S2 Tables).

Twenty-three percent of all plant species (18 spp, see S1 Table) had hemipterans feeding on them (13 spp., S3 Table), leading to networks with high specialization, high modularity, and low niche overlap among hemipterans (S4 Table). Those hemipteran species were tended by 12 ant species, but specialization and modularity were low in this layer (S4 Table). The most represented trophobionts were Aphididae (*Aphis spiraecola* and *Aphis fabae*) and Coccidae (*Parasaissetia nigra* and Coccidae sp 2), which together made up 77% of all interactions in the ant-trophobiont layer (S3 Table). The five most represented plant species in the ant-trophobiont layer lacked EFNs (S1 Table).

### Structure of the multilayer network

The multilayer network formed by the five interaction types had low but significant modularity, complementary specialization, weighted nestedness, and niche overlap among ants (Fig 1, Table 1). The structure of the ant-visit layer was similar to that of the complete network: low but significant modularity, complementary specialization, weighted nestedness, and low and non-significant niche overlap among ants (Table 1).

**Table 1:** Values for complementary specialization (H_2_’), modularity (Q), weighted nestedness (WNODF), niche overlap for ants (Horn), and their respective significances (P) for different layers in a multilayer ant-plant network.

Network	H_2_’	P (H_2_’)	Q	St. Q	WNODF	P (WNODF)	Horn	P (Horn)
Multilayer	0.27	0.001*	0.27	15.82*	27.12	0.001*	0.13	0.001*
Visit	0.26	0.001*	0.29	7.61*	22.01	0.004*	0.14	0.312
EFN	0.27	0.001*	0.30	4.37*	22.72	0.019*	0.26	0.002*
Flower	0.34	0.006*	0.51	2.80*	11.29	0.687	0.13	0.144
Tropho	0.45	0.001*	0.57	1.64	6.46	0.456	0.05	0.001*

* indicates significant differences

N = 999 randomizations, St. Q = standardized Q, Visit = ant-visit, EFN = ant-extrafloral nectar, Tropho = ant-trophobiont, Flower = ant-flower

**Fig 2:**
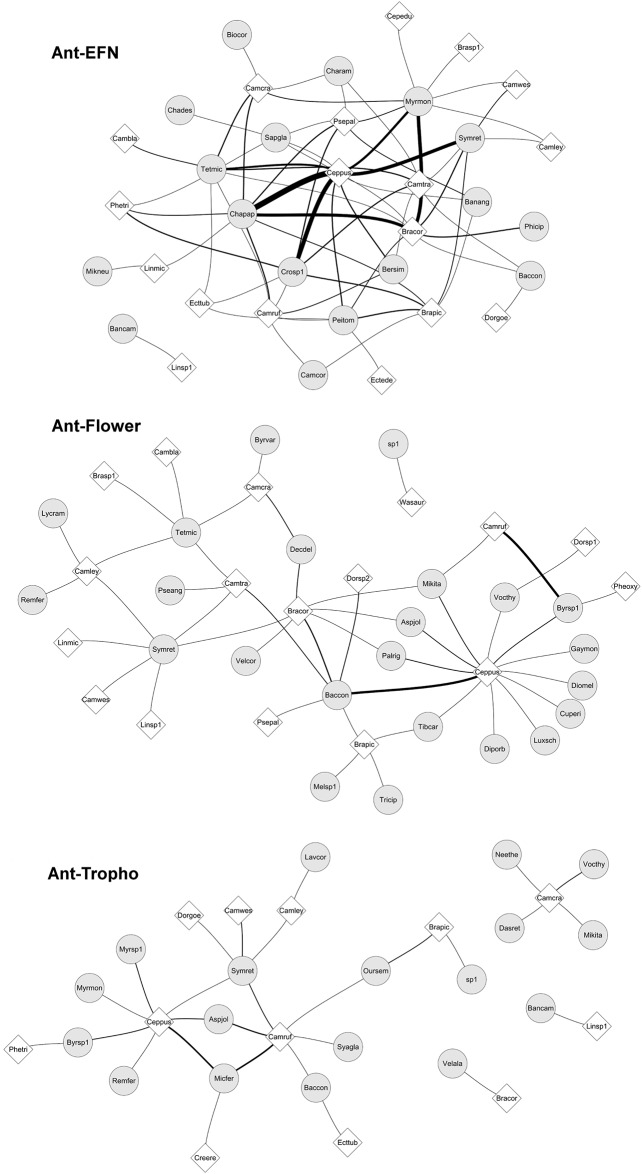
Network layers formed by interactions between ants and plants with extrafloral nectaries, trophobionts, and flowers. Circles represent plant species and diamonds represent ant species. Lines represent interactions between species and line thickness is proportional to interaction frequency. See ant and plant species codes in S1 and S2 Tables, respectively.

Considering the layers formed by different food sources, the ant-EFN layer had the lowest modularity and specialization, but the highest weighted nestedness and niche overlap among resource layers. The ant-flower layer had intermediate values for modularity, complementary specialization, weighted nestedness and niche overlap. The ant-trophobiont layer had the highest modularity and complementary specialization, but lower weighted nestedness and niche overlap among resource layers (Fig 2, Table 1).

### Differences among resource types

Consistent with our first expectation, the resource layers differed in network structure. The ant-EFN layer was the largest (GLM: deviance = 189.08, df = 2, p = 0.02, n = 7 sites), and had higher interaction richness (GLM: deviance = 23.94, df = 2, p = 0.001, n = 7 sites) and higher interaction frequency (GLM: deviance = 79.34, df = 2, p = 0.0003, n = 7 sites) than the ant-flower and ant-trophobiont layers, which were similar to one another (S5 Table). Similarly, the ant-flower and ant-trophobiont layers were similar in terms of network metrics (Table 2). The ant-flower layer was also similar to the ant-EFN layer in terms of specialization and niche partition metrics, but the ant-EFN layer exhibited lower complementary specialization than did the ant-trophobiont layer (Table 2).

**Table 2:** Structural comparison between resource types in the ant-plant multilayer network.

Structural metrics	Observed values for each layer		Differences among layers
	**EFN**	**Tropho**	**P-value**
Q	0.30	0.57	0.853
WNODF	22.72	6.46	0.974
H_2_’	0.27	0.45	0.032*
Horn	0.26	0.05	0.245
	**EFN**	**Flower**	**P-value**
Q	0.30	0.51	0.391
WNODF	22.72	11.29	0.984
H_2_’	0.27	0.34	0.571
Horn	0.26	0.13	0.083
	**Tropho**	**Flower**	**P-value**
Q	0.57	0.51	0.842
WNODF	6.46	11.29	0.688
H_2_’	0.45	0.34	0.839
Horn	0.05	0.13	0.803

* indicates significant differences

N = 999 randomizations, EFN = ant-extrafloral nectar layer, Tropho = ant-trophobiont layer, Flower = ant-flower layer

### The core formed by central species

**Fig 3:**
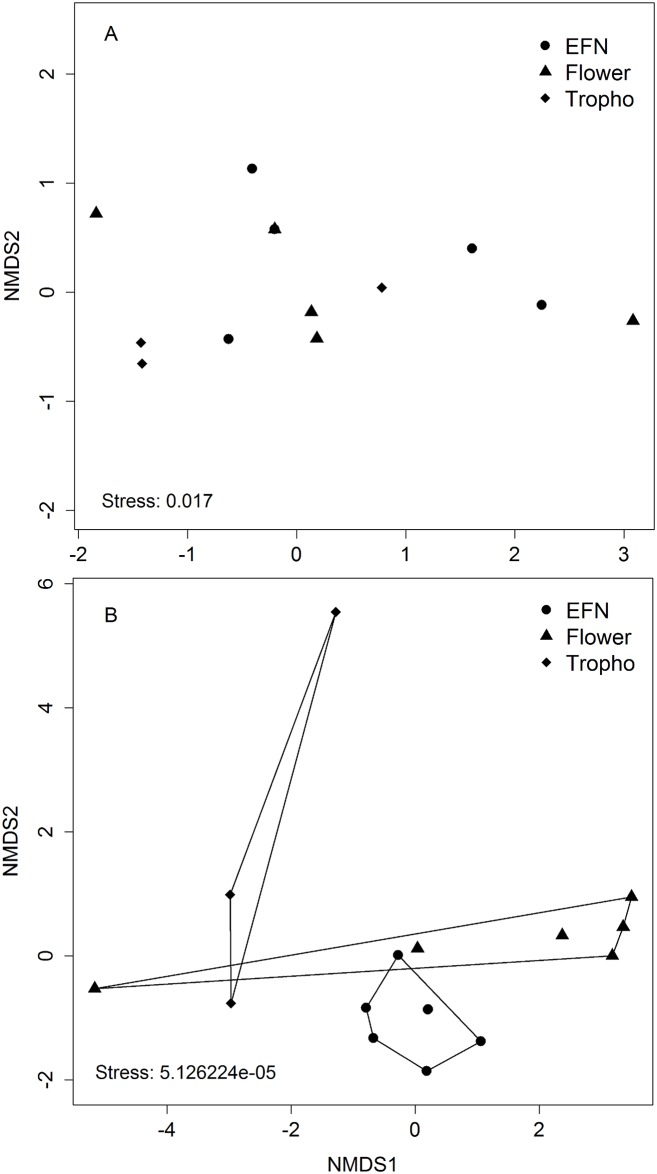
Nonmetric multidimensional scaling ordination (NMDS) showing the similarity of most central ant species (A), and central plant species (B) among resource layers in the multilayer ant-plant network. Points represent sampling sites and the polygons indicate significant differences (EFN = ant-extrafloral nectar, Flower = ant-flower, Tropho = ant-trophobiont).

The core of most central ant species in the ant-EFN layer was formed by eight ant species, followed by the ant-flower layer with five ant species, and the ant-trophobiont layer with four ant species. In total, the cores of resource layers were made up of nine ant species. Consistent with our second expectation, the species composition in these cores was similar (PERMANOVA: R^2^ = 0.145, p = 0.264, n = 7 sites, Fig 3A). Only *Cephalotes pusillus*, *Camponotus rufipes*, and *Camponotus crassus* occurred in the core of all resource layers. Together they made up 57% of all interactions in the multilayer network. In contrast to the high overlap in ant composition, the composition of the most central plant species in the cores of the resource layers were distinctly different (PERMANOVA: R^2^ = 0.226, p = 0.001; n = 7 sites, Fig 3B). Nineteen plant species formed the resource layer cores, with no species in common among them.

## Discussion

This study is the first to assess a multilayer network formed by ants feeding upon different food types in plants. The structure of this multilayer network follows no clear topological pattern corresponding to resource types, but when it is disentangled, interactions with distinct food sources can be seen to differ from one another in terms of specialization and resource partitioning among ants. Despite differing in structure, a core of a few ant species made up most of the interactions with resources provided by different plant species. Those findings suggest that the structural proprieties of interactions between ants and food rewards do not hold when different resource types are considered simultaneously. These results suggest a clear need to move from one single interaction to multiple types to understand communities. On the other hand, we show that the plant community is bound together by a few highly central ant species that could provide different ecological functions to plants. Below, we first discuss possible mechanisms underlying these findings, then conjecture how ant-food reward relationships can be understood from a network perspective.

Consistent with our first expectation, distinct resource types formed structurally different networks. EFN was the most common resource consumed by foliage-dwelling ants, followed by flower resources (pollen and/or nectar), and then hemipteran honeydew. Fruit, however, does not seem to be a common food source for ants in *campo rupestre* vegetation. In fact, EFNs attracted a larger number of ant species, promoted higher recruitment, and frequency of interactions than other food types (S2 and S5 Tables). EFNs also formed networks with the highest nestedness, highest niche overlap among ants, and largest ant core among resources, suggesting more interspecific co-occurrences in comparison to other resources. A nested pattern in ecological networks indicates the presence of a core in which frequent species interact with less frequent species in the network, leading to higher persistence and stability of the mutualism [59]. The ecological and evolutionary importance of EFNs for ant-plant community has been heavily discussed in literature [19,60]. Our results support the idea that EFNs are a key resource promoting ant diversity and driving the structure of the ant-plant community not only in savannas [61,62] and rainforests [23], but also in *campo rupestre*.

The higher specialization and non-nested pattern found in the ant-trophobiont layer would be expected if we consider that honeydew availability in *campo rupestre* is apparently more limited than nectar [25] and that honeydew, especially from Aphididae and Coccidae, is nitrogen-enriched and more nutritious than plant nectars [63]. As a consequence, it is likely that honeydew is a more valuable and rare resource for ants [23]. This seems likely to promote interspecific segregation, with superior competitors species dominating better quality resources [61]. In addition, the ant-trophobiont layer also had the lowest niche overlap among ants and the smallest core (four ant species) among food types, supporting the idea that honeydew promotes segregated patterns of ant species co-occurrence in *campo rupestre* [25]. Territorial competition among ants are well-known in tropical vegetation, where dominant ants organize interspecific interactions and drive community assembly [64].

Foraging on flowers involved similar ant diversity and frequency of interactions as did feeding on honeydew. Likewise, the structure of the ant-flower layer was very similar to the ant-trophobiont layer, both forming more specialized networks than found in the ant-EFN layer. Honeydew, floral nectar and pollen are resources of high quality and nutritional value for ants [65,66]. Since ants consume food rewards in opportunistic ways, it seems probable that resource availability and predictability across the year determine their foraging strategies: flowers normally are prevalent in the dry season, a period when trophobionts are less abundant [24,62]. This same phenological pattern might be taking place at our study site, where several plant species exhibit a flowering peak during the dry season [67]. Thus, is likely that ants switch food sources during the year, leading to a similar structure of interactions. On flowers, ants are typically considered robbers and thieves [68], although evidence that ants can also act as pollinators does exist [17]. Trophobionts are plant herbivores that reduce plant fitness, but when attended by ants might lead to indirect positive effects for plants (reviewed by [18]). Although we have not quantified interaction outcomes, our results illustrate how interactions that likely range from negative to positive effects are tied together in the community.

The core plant species belonged to different families and life forms [35], covering a wide spectrum of flower types, fruit types and secretory structure types. In contrast, a core of relatively few ant species made up most of the interactions with resources provided by different plant species, in line with our expectation. Three ant species stand out in the *campo rupestre*, since they were present in the cores of all resource networks. Although they encompass only 1% of the ant species recorded in the region [36], they are over-represented in the multilayer network (> 50% of records). Previous studies carried out in distinct habitats suggest that the cores of ant-EFN and ant-honeydew networks are composed by competitively superior ant species [25,31]. In fact, *Camponotus crassus* and *Camponotus rufipes* are numerically dominant and aggressive ants, which are considered truly trophobiont and plant mutualists in cerrado [69] and *campo rupestre* [70]. *Cephalotes pusillus* is a sub-dominant ant that has evolved some traits that favor its success on vegetation, such as a diet based largely on plant resources and a body morphology and a caste of soldiers specialized for nest defense [71]. We might expect that these species prevalence would reflect mostly their abundance. However, we verified that ant species centrality in this study is not influenced by their local abundance, but rather their recruitment rate (S6 Table). These evidence indicate that traits related to foraging strategies, such as massive recruitment and defense behavior, might explain the consistence of this core in different resource types.

In conclusion, we suggest that the structural patterns described so far for ant-plant networks are not consistent when interactions with multiple resources are considered. Ant interactions with EFNs, flowers and trophobionts formed networks that differed in ant diversity, specialization and niche overlap. However, a common core of a few ant species feed on these plant-derived food rewards, leading to a generalized multilayer network. This generalized structure mediated by a small core of ants may be a consequence of the opportunistic nature of ant-plant interactions [24]. On the other hand, foraging strategies of ant species appear to underlie the differences in specialization and niche partitioning in ant-plant interactions. These findings point to the importance of incorporating different types of interactions in order to unveil the complexity of communities. Whether the core species function as mutualists, antagonists or a combination is an open question that needs further investigation. These ant species might play a major ecological role in *campo rupestre*, as they appear to be involved in a diversity of ecosystem functions.

## Supporting Information

S1 Table Data on plant species and their interactions in the multilayer network (Species code = plant species code in the multilayer network; E = extrafloral nectaries, FL = flowers, FR = fruits, T = trophobionts, V = visits, Recruit = ant workers recruitment, symbol “-” indicates the absence of interaction with ants and absence of food resource). (PDF)

S2 Table Data on ant species and their interactions with different resource types in the multilayer network (Code = species code in the network, E = extrafloral nectaries, FL = flowers, FR = fruits, T = trophobionts, V = visits, Recruitment = ant workers recruitment). (PDF)

S3 Table Data on trophobionts and their interactions with plants and ants in the multilayer network. (PDF)

S4 Table Structural metrics performed for networks formed by interactions between plant species and trophobiont species (“Plant-Tropho”), and interactions between trophobiont species and ant species (“Tropho-Ant”) (symbol “*” indicates significant differences between observed value and Monte Carlo randomizations, n = 999, St. Q = standardized Q value). (PDF)

S5 Table General properties of the networks formed by interactions between ants and different food types in the multilayer network. Values are presented as average ± standard deviation for each layer (EFN = extrafloral nectar, Tropho = trophobiont).
(PDF)

S6 Table Generalized linear model (GLM) analysis showing the relationship between centrality degree of ant species (n = 30) and their abundance and recruitment. Abundance data was recorded by pitfall traps installed in the same seven studied sites (symbol “*” represents significant differences, Df = degrees of freedom).
(PDF)
