# Supplementary material for: Few Ant Species Play a Central Role Linking Different Plant Resources in a Network in Rupestrian Grasslands
Source: PLoS One. 2016 Dec 2;11(12):e0167161. doi: 10.1371/journal.pone.0167161 (PMC5135051; doi:10.1371/journal.pone.0167161)
Supplement: S1 Table — (PDF) [file pone.0167161.s001.pdf]

S1 Table. Data on plant species and their interactions in the multilayer network (Species code = plant species code in the multilayer network; E = extrafloral nectaries, FL = flowers, FR = fruits, T = trophobionts, V = visits, rich = richness, recruit = ant workers recruitment, symbol "-" indicates the absence of interaction with ants and absence of food resource).

Plant taxa	Species code	Plant abundance	Proportion of interacting plants (%)	Interaction frequency	Resource type					Ant rich/ species	Ant recruit/ species
**Acanthaceae**											
Ruellia vilosa	Ruevil	17	5.9	1	-	-	-	-	V	1	1
**Apocynaceae**											
Oxypetalum sp1	-	2	0	-	-	-	-	-	-	-	-
**Araceae**											
Philodendron cipoense	Phicip	3	100.0	14	E	-	-	-	V	4	29
**Arecaceae**											
Syagrus glaucescens	Syagla	8	87.5	18	-	-	-	T	V	2	46
Syagrus pleioclada	-	4	0	-	-	-	-	-	-	-	-
**Asteraceae**											
Acritopappus confertus	-	4	0	-	-	-	-	-	-	-	-
Aspilia jolyana	Aspjol	91	19.8	22	-	FL	-	T	V	5	46
Asteraceae sp1	-	1	0	-	-	-	-	-	-	-	-
Baccharis concinna	Baccon	44	40.9	47	E	FL	-	T	V	12	96
Chromolaena sp1	Chrsp1	7	28.6	2	-	-	-	-	V	1	2

Plant taxa	Species code	Plant abundance	Proportion of interacting plants (%)	Interaction frequency	Resource type					Ant rich/ species	Ant recruit/ species
Dasyphyllum reticulatum	Dasret	18	11.1	2	-	-	-	T	V	2	21
Echinocoryne schwenkiifolia	Echsch	9	11.1	1	-	-	-	-	V	1	1
Lessingianthus warmingianum	Leswar	2	50.0	1	-	-	-	-	V	1	7
Lychnophora ramosissima	Lycpas	46	30.4	18	-	FL	-	-	V	7	26
Lychnophora rupestris	Lyctri	4	50.0	2	-	-	-	-	V	2	6
Mikania itambana	Mikita	28	35.7	14	-	FL	-	T	V	4	46
Mikania neurocaula	Mikneu	2	50.0	1	E	-	-	-	-	1	1
Porophyllum angustissimum	-	7	-	-	-	-	-	-	-	-	-
Pseudobrickellia angustissima	Pseang	2	50	1	-	FL	-	-	-	1	1
Symphyopappus reticulatus	Symret	53	47.2	60	E	FL	-	T	V	11	111
**Aquifoliaceae**											
Ilex theezans	-	2	0	-	-	-	-	-	-	-	-
**Cactaceae**											
Pilosocereus cipoense	Pilcip	2	50.0	1	-	-	-	-	V	1	1
**Clusiaceae**											
Kielmeyera petiolaris	Kiepet	3	100.0	3	-	-	-	-	V	2	3
Kielmeyera regalis	Kiereg	4	25.0	1	-	-	-	-	V	1	1
**Dilleniaceae**											
Davila elliptica	Davell	3	100.0	6	-	-	-	-	V	5	15
**Ericaceae**											

Plant taxa	Species code	Plant abundance	Proportion of interacting plants (%)	Interaction frequency	Resource type					Ant rich/ species	Ant recruit/ species
Gaylussacia montana	Gaymon	10	50.0	5	-	FL	-	-	V	3	8
**Eriocaulaceae**											
Paepalanthus vellozioides	Paevel	4	75.0	8	-	-	-	-	V	4	13
**Erythroxylaceae**											
Erythroxylum campestris	Erycam	16	25.0	5	-	-	-	-	V	4	6
**Euphorbiaceae**											
Bernardia similis	Bersim	1	100.0	6	E	-	-	-	-	3	14
Croton sp1	Crosp1	14	57.1	31	E	-	-	-	V	7	49
Euphorbiaceae sp1	-	2	0	-	-	-	-	-	-	-	-
Sapium glandulatum	Sapgla	3	66.7	7	E	-	-	-	V	5	19
**Fabaceae**											
Bionia coriacea	Biocor	12	50.0	6	E	-	-	-	V	5	16
Chamaecrista cipoana	Chacip	11	27.3	3	-	-	-	-	V	3	3
Chamaecrista desvauxii	Chades	2	50.0	2	E	-	-	-	V	2	2
Chamaecrista papillata	Chapap	12	108.3	58	E	-	-	-	V	10	58
Chamaecrista ramosa	Charam	11	54.5	11	E	-	-	-	V	6	11
Dalbergia miscolobuim	Dalmis	6	66.7	4	-	-	-	-	V	2	4
Mimosa maguirei	-	1	0	-	-	-	-	-	-	-	-
**Lamiaceae**											
Eriope hypoleuca	Erihyp	8	25.0	3	-	-	-	-	V	2	3

Plant taxa	Species code	Plant abundance	Proportion of interacting plants (%)	Interaction frequency	Resource type					Ant rich/ species	Ant recruit/ species
Hypenia macrantha	-	3	0	-	-	-	-	-	-	-	-
Hyptis proteoides	-	7	0	-	-	-	-	-	-	-	-
Hyptis sp1	Hypsp1	30	16.7	7	-	-	-	-	V	4	8
Lamiaceae sp1	Lamsp1	3	100.0	3	-	-	-	-	V	3	3
Lamiaceae sp2	-	1	0	-	-	-	-	-	-	-	-
Lamiaceae sp3	-	2	0	-	-	-	-	-	-	-	-
**Lauraceae**											
Ocotea langsdorffii	-	1	0	-	-	-	-	-	-	-	-
**Loganiaceae**											
Antonia ovata	-	1	0	-	-	-	-	-	-	-	-
Spigelia sellowiana	Spisel	5	20.0	1	-	-	-	-	V	1	1
**Lythraceae**											
Cuphea ericoides	Cuperi	29	20.7	8	-	FL	-	-	V	3	10
Diplusodon hirsutus	-	5	0	-	-	-	-	-	-	-	-
Diplusodon orbicularis	Diporb	32	12.5	4	-	FL	-	-	V	3	4
**Malpighiaceae**											
Banisteriopsis angustifolia	Banang	9	55.6	9	E	-	-	-	V	5	19
Banisteriopsis campestris	Bancam	1	100.0	2	E	-	-	T	-	1	10
Byrsonima sp1	Byrsp1	19	78.9	31	-	FL	FR	T	V	9	51
Byrsonima vacciniifolia	Byrvar	12	41.7	7	-	FL	FR	-	V	4	14

Plant taxa	Species code	Plant abundance	Proportion of interacting plants (%)	Interaction frequency	Resource type					Ant rich/ species	Ant recruit/ species
Malpighiaceae sp1	-	1	0	-	-	-	-	-	-	-	-
Peixotoa tomentosa	Peitom	12	66.7	15	E	-	-	-	V	6	71
Tetrapterys microphylla	Tetmic	36	50.0	26	E	FL	-	-	V	10	33
**Malvaceae**											
Byttneria sp1	-	3	0	-	-	-	-	-	-	-	-
**Melastomataceae**											
Lavoisiera confertiflora	Lavcon	1	100.0	2	-	-	-	-	V	2	2
Lavoisiera cordata	Lavcor	14	7.1	1	-	-	-	T	-	1	4
Lavoisiera sp1	Melsp2	8	12.5	1	-	-	-	-	V	1	1
Marcetia taxifolia	-	15	0	-	-	-	-	-	-	-	-
Melastomataceae sp1	Melsp1	3	66.7	2	-	FL	-	-	V	2	2
Melastomataceae sp2	-	1	0	-	-	-	-	-	-	-	-
Melastomataceae sp3	-	5	0	-	-	-	-	-	-	-	-
Miconia ferruginata	Micfer	2	100.0	9	-	-	-	T	-	3	98
Microlicia fulva	Micful	16	18.8	3	-	-	-	-	V	2	3
Microlicia sp1	-	3	0	-	-	-	-	-	-	-	-
Microlicia tetrasticha	-	1	0	-	-	-	-	-	-	-	-
Tibouchina cardinalis	Tibcar	5	20.0	2	-	FL	-	-	-	2	2
Tibouchina heteromalla	-	1	0	-	-	-	-	-	-	-	-
Trembleya glandulosa	-	1	0	-	-	-	-	-	-	-	-

Plant taxa	Species code	Plant abundance	Proportion of interacting plants (%)	Interaction frequency	Resource type					Ant rich/ species	Ant recruit/ species
Trembleya laniflora	Trelan	11	45.5	5	-	-	-	-	V	3	5
**Myrsinaceae**											
Myrsine monticola	Myrmon	11	100.0	46	E	-	-	T	V	11	100
**Myrtaceae**											
Campomanesia pubescens	-	1	0	-	-	-	-	-	-	-	-
Myrcia sp1	Myrsp1	31	16.1	6	-	-	-	T	V	3	8
**Nyctaginaceae**											
Guapira areolata	-	1	0	-	-	-	-	-	-	-	-
Guapira noxia	Guanox	3	66.7	3	-	-	-	-	V	2	3
Neea theifera	Neethe	10	50.0	9	-	-	-	T	V	3	13
**Ochnaceae**											
Luxemburgia schwackeana	Luxsch	1	100.0	1	-	FL	-	-	-	1	7
Luxemburgia villosa	-	3	0	-	-	-	-	-	-	-	-
Ouratea semiserrata	Oursem	13	46.2	13	-	-	-	T	V	4	15
**Polygonaceae**											
Coccoloba acrostichoides	Cocacr	6	83.3	6	-	-	-	-	V	1	7
Coccoloba cereifera	Coccer	1	100.0	1	-	-	-	-	V	1	3
**Proteaceae**											
Roupala montana	Roumon	3	33.3	3	-	-	-	-	V	2	3
**Rubiaceae**											

Plant taxa	Species code	Plant abundance	Proportion of interacting plants (%)	Interaction frequency	Resource type					Ant rich/ species	Ant recruit/ species
Declieuxia deltoidea	Decdel	3	66.7	4	-	FL	-	-	-	2	8
Declieuxia fruticosa	-	4	0	-	-	-	-	-	-	-	-
Dioidia mello-barretoi	Diomel	1	200.0	2	-	FL	-	-	V	1	2
Palicourea rigida	Palrig	8	37.5	5	-	FL	-	-	V	2	12
Remijia ferruginea	Remfer	9	77.8	14	-	FL	FR	T	V	5	26
Rubiaceae sp1	-	1	0	-	-	-	-	-	-	-	-
Sabiceae brasiliensis	Sabbra	2	50.0	1	-	-	-	-	V	1	2
**Trigoniaceae**											
Trigonia cipoensis	Tricip	49	10.2	6	-	FL	-	-	V	4	11
**Unidentified**											
sp1	sp1	2	50.0	3	-	FL	-	T	V	2	6
**Velloziaceae**											
Barbacenia flava	Barfla	39	59.0	46	-	-	-	-	V	12	60
Vellozia alata	Velala	12	50.0	12	-	-	-	T	V	5	95
Vellozia cf. aloifolia	Velniv	5	60.0	3	-	-	-	-	V	2	3
Vellozia nanuzae	Velcor	2	50.0	2	-	-	-	-	V	2	2
Vellozia nivea	Velnan	43	55.8	47	-	-	FR	-	V	13	146
Vellozia varabilillis	Velsp1	31	64.5	33	-	FL	-	-	V	11	76
**Verbenaceae**											
Lippia florida	Lipflo	15	6.7	1	-	-	-	-	V	1	1

Plant taxa	Species code	Plant abundance	Proportion of interacting plants (%)	Interaction frequency	Resource type					Ant rich/ species	Ant recruit/ species
Lippia sp1	Lipsp1	6	50.0	4	-	-	-	-	V	2	4
**Vochysiaceae**											
Qualea cordata	Quacor	1	100.0	2	-	-	-	-	V	2	2
Vochysia elliptica	-	5	0	-	-	-	-	-	-	-	-
Vochysia thyrsoidea	Vocthy	14	64.3	19	-	FL	-	T	V	6	57
