# Supplementary material for: Few Ant Species Play a Central Role Linking Different Plant Resources in a Network in Rupestrian Grasslands
Source: PLoS One. 2016 Dec 2;11(12):e0167161. doi: 10.1371/journal.pone.0167161 (PMC5135051; doi:10.1371/journal.pone.0167161)
Supplement: S2 Table — (PDF) [file pone.0167161.s002.pdf]

**S2 Table. Data on ant species and their interactions with different resource types in the multilayer network (Code = species code in the network, E = extrafloral nectaries, FL = flowers, FR = fruits, T = trophobionts, V = visits, Recruitment = ant workers recruitment).**

| Ant taxa | Species code | Interaction frequency | | | | | Recruitment | Recruitment / plant (mean ± SD) | Partner richness |
|---|---|---|---|---|---|---|---|---|---|
| | | E | FL | FR | T | V | | | |
| **Formicidae** | | | | | | | | | |
| **Dolichoderinae** | | | | | | | | | |
| *Dorymyrmex goeldii* | Dorgoe | 1 | - | - | 1 | 1 | 3 | 1 | 3 |
| *Dorymyrmex sp1* | Dorsp1 | - | 1 | - | - | 4 | 6 | 1.5 ± 1 | 4 |
| *Dorymyrmex sp2* | Dorsp2 | - | 2 | - | - | 6 | 14 | 2 ± 0.8 | 5 |
| *Linepithema micans* | Linmic | 2 | 1 | - | - | 5 | 15 | 1.9 ± 2.1 | 7 |
| *Linepithema sp1* | Linsp1 | 1 | 1 | - | 1 | - | 11 | 5.5 ± 6.4 | 2 |
| **Ectatomminae** | | | | | | | | | |
| *Ectatomma edentatum* | Ectede | 1 | - | - | - | 1 | 2 | 2 | 1 |
| *Ectatomma tuberculatum* | Ecttub | 3 | - | - | 1 | 6 | 27 | 3.9 ± 4.2 | 7 |
| **Formicinae** | | | | | | | | | |
| *Brachymyrmex cordemoyi* | Bracor | 28 | 10 | - | 1 | 55 | 388 | 5.7 ± 11.0 | 30 |
| *Brachymyrmex pictus* | Brapic | 12 | 4 | 1 | 3 | 25 | 154 | 3.9 ± 8.0 | 21 |
| *Brachymyrmex sp1* | Brasp1 | 1 | 1 | - | - | 2 | 5 | 1.3 ± 0.5 | 3 |
| *Camponotus blandus* | Cambla | 2 | 1 | - | - | 8 | 19 | 2.4 ± 1.6 | 5 |
| *Camponotus crassus* | Camcra | 10 | 4 | 2 | 5 | 59 | 150 | 2.6 ± 3.5 | 29 |
| *Camponotus leydigi* | Camley | 2 | 4 | - | 2 | 4 | 24 | 2 ± 1.2 | 9 |

| Ant taxa | Species code | Interaction frequency | | | | | Recruitment | Recruitment / plant (mean ± SD) | Partner richness |
|---|---|---|---|---|---|---|---|---|---|
| | | E | FL | FR | T | V | | | |
| *Camponotus rufipes* | Camruf | 9 | 7 | 3 | 12 | 50 | 205 | 3.1 ± 8.4 | 36 |
| *Camponotus trapeziceps* | Camtra | 10 | 5 | - | - | 23 | 44 | 1.3 ± 0.8 | 19 |
| *Camponotus vitatus* | Camvit | - | - | - | - | 1 | 1 | 1 | 1 |
| *Camponotus westermanni* | Camwes | 3 | 1 | - | 2 | 17 | 42 | 2.3 ± 2.5 | 10 |
| **Myrmicinae** | | | | | | | | | |
| *Atta laevigata* | Attlae | - | - | - | - | 1 | 9 | 9 | 1 |
| *Cephalotes eduarduli* | Cepedu | 1 | - | - | - | 1 | 5 | 5 | 1 |
| *Cephalotes pusillus* | Ceppus | 54 | 21 | 2 | 13 | 204 | 544 | 2.8 ± 3.3 | 51 |
| *Crematogaster crinosa* | Crecri | - | - | - | - | 1 | 1 | 1 | 1 |
| *Crematogaster erecta* | Creere | - | - | - | 1 | 1 | 10 | 5 | 2 |
| *Nesomyrmex sp1* | Nessp1 | - | - | - | - | 1 | 1 | 1 | 1 |
| *Nesomyrmex spininodis* | Nesspi | - | - | - | - | 3 | 7 | 2.3 ± 2.3 | 3 |
| *Pheidole oxyops* | Pheoxy | - | 1 | - | - | 1 | 2 | 1 | 2 |
| *Pheidole triconstricta* | Phetri | 6 | - | - | 1 | 8 | 32 | 2.3 ± 2.1 | 9 |
| *Wasmannia auropunctata* | Wasaur | - | 1 | - | - | - | 4 | 4 | 1 |
| **Pseudomyrmecinae** | | | | | | | | | |
| *Pseudomyrmex gracilis* | Psegra | - | - | - | - | 2 | 2 | 1 | 2 |
| *Pseudomyrmex pallidus* | Psepal | 12 | 1 | - | - | 28 | 42 | 1.2 ± 0.6 | 19 |
| *Pseudomyrmex termitarius* | Pseter | - | - | - | - | 1 | 1 | 1 | 1 |
| **Total** | | **158** | **66** | **8** | **43** | **519** | **1770** | | |
