# Supplementary material for: Few Ant Species Play a Central Role Linking Different Plant Resources in a Network in Rupestrian Grasslands
Source: PLoS One. 2016 Dec 2;11(12):e0167161. doi: 10.1371/journal.pone.0167161 (PMC5135051; doi:10.1371/journal.pone.0167161)
Supplement: S3 Table — (PDF) [file pone.0167161.s003.pdf]

S3 Table. Data on trophobionts and their interactions with plants and ants in the multilayer network.

Trophobiont taxa	Frequency in plants	Ant species interacting	Ant workers/ plant	Total ants recruitment
**Aphididae**				
Aphis fabae	3	3	12 ± 8.5	36
Aphis spiraecola	5	4	17.2 ± 29	103
**Coccidae**				
Coccidae sp1	1	1	1	1
Coccidae sp2	6	4	4.5 ± 8.1	27
Parasaissetia nigra	7	5	3.4 ± 2.7	24
**Margarodidae**				
Margarodidae sp1	2	2	3.5 ± 0.5	7
**Unidentified family**				
Hemiptera sp1	1	1	1	1
Hemiptera sp2	1	2	1	2
Hemiptera sp3	1	1	7	7
Hemiptera sp4	1	1	1	1
Hemiptera sp5	1	1	2	2
Hemiptera sp6	1	1	1	1
