# Supplementary material for: Few Ant Species Play a Central Role Linking Different Plant Resources in a Network in Rupestrian Grasslands
Source: PLoS One. 2016 Dec 2;11(12):e0167161. doi: 10.1371/journal.pone.0167161 (PMC5135051; doi:10.1371/journal.pone.0167161)
Supplement: S4 Table — (PDF) [file pone.0167161.s004.pdf]

S4 Table. Structural metrics performed for networks formed by interactions between plant species and trophobiont species ("Plant-Tropho"), and interactions between trophobiont species and ant species ("Tropho-Ant") (symbol "*" indicates significant differences between observed value and Monte Carlo randomizations, n=999, St. Q = standardized Q value).

Network type	Metric	Real value	Significance
**Plant-Tropho**	WNODF	2.21	p = 0.001*
	H2'	0.73	p = 0.001*
	Niche overlap	0.09	p = 0.001*
	Modularity	0.70	St. Q = 22.162*
**Tropho-Ant**	WNODF	15.34	p = 0.142
	H2'	0.28	p = 0.929
	Niche overlap	0.17	p = 0.049*
	Modularity	0.38	St. Q = 0.635
