# Supplementary material for: Few Ant Species Play a Central Role Linking Different Plant Resources in a Network in Rupestrian Grasslands
Source: PLoS One. 2016 Dec 2;11(12):e0167161. doi: 10.1371/journal.pone.0167161 (PMC5135051; doi:10.1371/journal.pone.0167161)
Supplement: S5 Table — Values are presented as average ± standard deviation for each layer (EFN = extrafloral nectar, Tropho = trophobiont). (PDF) [file pone.0167161.s005.pdf]

S5 Table. General properties of the networks formed by interactions between ants and different food types in the multilayer network. Values are presented as average ± standard deviation for each layer (EFN = extrafloral nectar, Tropho = trophobiont).

Layers	Size	Richness	Frequency
Ant-EFN	69.71 ± 40.89	11.85 ± 4.33	22.57 ± 14.16
Ant-Flower	41.28 ± 34.65	7.28 ± 3.86	9.42 ± 5.62
Ant-Tropho	21.57 ± 24.01	4.57 ± 2.76	6.14 ± 4.25
