# Supplementary material for: Few Ant Species Play a Central Role Linking Different Plant Resources in a Network in Rupestrian Grasslands
Source: PLoS One. 2016 Dec 2;11(12):e0167161. doi: 10.1371/journal.pone.0167161 (PMC5135051; doi:10.1371/journal.pone.0167161)
Supplement: S6 Table — Abundance data was recorded by pitfall traps installed in the same seven studied sites (symbol “*” represents significant differences, Df = degrees of freedom). (PDF) [file pone.0167161.s006.pdf]

S6 Table. Generalized linear model (GLM) analysis showing the relationship between centrality degree of ant species (n = 30) and their abundance and recruitment. Abundance data was recorded by pitfall traps installed in the studied sites (symbol "*" represents significant differences, Df = degrees of freedom).

Response variable	Explanatory variable	Df	Deviance	P-value	Error distribution
Centrality degree	Ant recruitment	28	55.358	< 0,001*	Negative binomial
Centrality degree	Ant abundance	28	0.10802	0.7424	Poisson
